# 16S rRNA amplicon sequencing of seawater microbiota from Rayong Province, Thailand, affected by oil spills

**DOI:** 10.1128/mra.00966-23

**Published:** 2024-03-01

**Authors:** Komkiew Pinpimai, Porntep Punnarak, Tongchai Thitiphuree, Se Songploy, Arnupap Panichpol, Anchalee Chankong, Suchana Chavanich, Voranop Viyakarn

**Affiliations:** 1 Aquatic Resources Research Institute, Chulalongkorn University, Bangkok, Thailand; 2 Department of Marine and Coastal Resources, Bangkok, Thailand; 3 Reef Biology Research Group, Department of Marine Science, Faculty of Science, Chulalongkorn University, Bangkok, Thailand; California State University San Marcos, San Marcos, California, USA

**Keywords:** 16S rRNA, microbiota, seawater, oil spills, Thailand

## Abstract

We reported on microbial communities isolated from 18 seawater samples affected by oil spills in Rayong province, Thailand, using the V3-V4 region of 16S rRNA gene sequencing.

## ANNOUNCEMENT

Oil spills can cause significant problems to marine ecosystems due to introducing toxic compounds such as hydrocarbons, heavy metals, and dispersants (1, 2). These compounds can disrupt the balance of microbial communities, leading to significant ecological changes (1, 3). Some microorganisms can break down hydrocarbons and use them for energy, but the response of microbial communities to oil spills is complex and depends on many factors (2). Here, we reported on the microbial communities found in 18 seawater samples during oil spills in Rayong province, Thailand, in early 2022.

A total of 18 seawater (10 L per sample) samples were collected in triplicate on 27 January 2022, 2 days after the report of the spill event. The selection of sampling sites was determined by the wind direction: at the site of the spill itself; 1 km southeast, southwest, and northwest of the spill; 2 km northeast of the spill; and 14 km northeast of the spill, as oil was observed in this area. Water samples, 10 L per sample, from each location and depth, were individually filtered through 0.45-µm pore size filters (PALL GN-6 Metricel) within 24  h and were stored, chilled (4°C), and shipped to the laboratory within 48 h for DNA extraction. For each collection site and depth, the filtrate from the triplicate samples was pooled before DNA extraction. DNA was extracted from filtered papers using a Blood & Tissue DNA Isolation Kit, according to the manufacturer’s instructions. The DNA was sent to Macrogen, Inc (Seoul, South Korea) for sequencing. The DNA was amplified using the V3-V4 region of the 16S rRNA gene with the forward primer CCTACGGGNGGCWGCAG and the reverse primer GACTACHVGGGTATCTAATCC. A fragment library was prepared using a Herculase II Fusion DNA Polymerase Nextera XT Index V2 Kit. Paired-end reads (2 × 100 bp) were obtained from a MiSeq instrument (Illumina, Inc, San Diego, CA, US). Raw reads were processed in R studio (Version 2023.06.0+421) using DADA2 (v1.18) package (4) using default parameters. Operational taxonomic units were generated via open reference picking and identified and analyzed using the SILVA nr99 database (v138.1) (5). Sample and sequence data are summarized in Table 1.

**TABLE 1:** Descriptions of seawater samples collected during oil spills

Sample ID	Lab ID	Depth^*a*^	SRA accession no.	Location		Total bases (bp)	Total reads	GC (%)
13J031	OSR0_3_1	3	SRR24954508	N12° 29' 17.9"	E101° 11' 48.0"	42,410,298	140,898	53
05J111	OSR1_1_1	1	SRR24954499	N12° 30' 03.9"	E101° 12' 34.9"	33,582,570	111,570	53.4
04J211	OSR2_1_1	1	SRR24954500	N12° 29' 37.5"	E101° 12' 09.5"	25,122,062	83,462	52.5
03J221	OSR2_2_1	2	SRR24954501	N12° 29' 37.5"	E101° 12' 09.5"	35,760,606	118,806	52.6
02J311	OSR3_1_1	1	SRR24954511	N12° 29' 04.6"	E101° 12' 12.7"	33,628,924	111,724	53
01J321	OSR3_2_1	2	SRR24954512	N12° 29' 04.6"	E101° 12' 12.7"	44,070,012	146,412	52.6
08J51	OSR5_1_1	1	SRR24954496	N12° 29' 37.8"	E101° 11' 23.3"	32,877,026	109,226	53.2
07J52	OSR5_2_1	2	SRR24954497	N12° 29' 37.8"	E101° 11' 23.3"	34,237,546	113,746	52.9
06J53	OSR5_3_1	3	SRR24954498	N12° 29' 37.8"	E101° 11' 23.3"	36,693,104	121,904	52.9
18J01	OSR_0_1	1	SRR24954503	N12° 29' 17.9"	E101° 11' 48.0"	32,732,546	108,746	53.3
17J02	OSR_0_2	2	SRR24954504	N12° 29' 17.9"	E101° 11' 48.0"	29,080,212	96,612	53.3
16J12	OSR_1_2	2	SRR24954505	N12° 30' 03.9"	E101° 12' 34.9"	27,807,584	92,384	53.2
15J13	OSR_1_3	3	SRR24954506	N12° 30' 03.9"	E101° 12' 34.9"	29,349,306	97,506	53.4
11J41	OSR_4_1	1	SRR24954510	N12° 28' 59.5"	E101° 11' 19.4"	34,841,954	115,754	52.9
10J42	OSR_4_2	2	SRR24954494	N12° 28' 59.5"	E101° 11' 19.4"	32,901,106	109,306	53.1
09J43	OSR_4_3	3	SRR24954495	N12° 28' 59.5"	E101° 11' 19.4"	39,304,580	130,580	52.5
14J31	OSR_3_1	0	SRR24954507	N12° 29' 04.6"	E101° 12' 12.7"	31,151,694	103,494	52.7
12J32	OSR_3_2	3	SRR24954509	N12° 29' 04.6"	E101° 12' 12.7"	36,954,974	122,774	53.3

^*a*^ Depth: 0, water surface on which oil spills were visible; 1, 1 m below water surface; 2, middle between the water surface and deepest point; 3, the deepest point.

Based on the amplicon analysis, the phylum relative abundance in all samples ranged from Proteobacteria, Cyanobacteria, Bacteroidota, and Actinobacteriota, respectively.

Here is the report revealing the seawater microbial community affected by oil spills in Rayong province, Thailand.

## Data Availability

All data are available through NCBI BioProject ID PRJNA984631. Individual accessions for each sample can be found in Table 1.
